# American Medical Society in Paris

**Published:** 1852-07

**Authors:** 


*American Medical Society in Paris.*—The number of Americans resorting to Paris for pursuits connected with medical science, is so large that a society has lately been organized, the objects of which are briefly set forth in the accompanying communication made at the last meeting of the Am. Med. Association, by a delegation from the new society. These objects must at once commend themselves to the cordial approbation of the profession in this country. Such an organization, aside from other advantages, will prove highly useful to our countrymen in Paris, by facilitating the special purposes of their residence in that city. We have received a letter from the secretary of the society, which we hereby acknowledge. It will afford us pleasure to send the Buffalo Journal, as desired, and to contribute, in any mode within our power, to the success of the undertaking. The communication above referred to is as follows:

The undersigned, delegates of the "American Medical Society in Paris," beg leave to submit a few remarks upon the origin, intention and present condition of this institution.

This society, convened for the first time in the month of November, 1851, has for its object the bringing together of American medical men residing in Paris, and the consequent diffusion of American medical and scientific knowledge among them. This institution, as yet in its infancy, has been sanctioned by the French government, and already recognized as a society by the numerous institutions of a similar character in the city. It numbers already fifty regular, active, besides a number of honorary and privileged members. The society, when it becomes more permanently fixed, will undoubtedly publish a journal, containing, in addition to its own original articles, the most interesting foreign medical news of the day, and from the nature of its position, must promise great advantages to the American practitioner in the United States. It most respectfully requests the usual interchanges of the numerous and able medical and scientific journals in the United States.

Circulars requesting this favor have already been addressed to the editors of their respective journals, and the society flatters itself that their request will not be in vain.

The library and reading room attached to the society being open to scientific gentlemen of all nations, will be the means of more thoroughly diffusing American medical literature, and correcting numerous absurd ideas prevailing abroad with regard to our scientific institutions and general attainments.

ALEXANDER J. SEMMES, M. D.,
WM. H. BERRY, M. D.,
R. M. JONES, M. D.,
*Delegation of American Medical Society in Paris.*

Paris, March 27th, 1852.

